# Significance of fractional exhaled nitric oxide in chronic eosinophilic pneumonia: a retrospective cohort study

**DOI:** 10.1186/1471-2466-14-81

**Published:** 2014-05-12

**Authors:** Ji Young Park, Taehoon Lee, Hongyeul Lee, Yeon Joo Lee, Jong Sun Park, Young-Jae Cho, Ho Il Yoon, Jae Ho Lee, Choon-Taek Lee

**Affiliations:** 1 Division of Pulmonary and Critical Care Medicine, Department of Internal Medicine and Respiratory Center, Seoul National University Bundang Hospital, 173-82 Gumi-Ro, Bundang-Gu, Seongnam 463-707, Republic of Korea; 2 Division of Pulmonary and Critical Care Medicine, Department of Internal Medicine, Seoul National University College of Medicine, Seoul, Republic of Korea

**Keywords:** Chronic eosinophilic pneumonia, Fractional exhaled nitric oxide, Biomarker, Corticosteroid

## Abstract

**Background:**

Chronic eosinophilic pneumonia (CEP) is characterized by chronic eosinophilic infiltration of the lung. It is dramatically responsive to corticosteroid treatment, but symptoms and radiopacities recur frequently after tapering or discontinuing the medication. Fractional exhaled nitric oxide (FeNO) is a well-known noninvasive marker of eosinophilic airway inflammation. The aim of this retrospective cohort study was to investigate the relationships of FeNO with peripheral eosinophilia and the clinical state of CEP and its validity for predicting exacerbation of CEP.

**Methods:**

Standard clinical and laboratory parameters, peripheral eosinophil percentage and count, and FeNO level were measured in 18 patients with CEP at several assessment points over 1 year.

**Results:**

FeNO level was positively correlated with peripheral eosinophil count (*r* = 0.341, *P* = 0.005) and percentage (*r* = 0.362, *P* = 0.003). The median (IQR) FeNO levels were 79 (41–88) and 35 (26–49) ppb in uncontrolled (13/74 measurements) and controlled (61/74 measurements) CEP, respectively (*P* = 0.010). The FeNO level of 66.0 ppb showed the largest area under the curve (0.835) for predicting exacerbation of CEP (sensitivity = 0.80, specificity = 0.84).

**Conclusion:**

FeNO may be useful for monitoring eosinophilic parenchymal inflammation and determining the appropriate corticosteroid dose in CEP.

## Background

Chronic eosinophilic pneumonia (CEP) is a rare disease of unknown cause. It is characterized by chronic respiratory symptoms, bilateral peripheral lung opacities, pulmonary eosinophilia, and/or peripheral eosinophilia. CEP shows a dramatic response to corticosteroids. Symptoms and radiopacities resolve rapidly after corticosteroid treatment [1], but they recur frequently after tapering or discontinuing the medication [1,2]. Most patients need prolonged tailored treatment, similar to those with asthma [3]. Therefore, a marker is required to assist in monitoring and controlling CEP.

FeNO is an important marker of eosinophilic airway inflammation in diseases such as asthma and nonasthmatic eosinophilic bronchitis [4]. In asthma, FeNO level is significantly correlated with eosinophil counts in bronchoalveolar lavage (BAL) fluid, induced sputum, and airway mucosal tissue [5-7]. It can identify patients with asthma who are likely to benefit from corticosteroid treatment and have reduced exacerbation rates [8-10]. Transition of asthma from the well-controlled to the poorly controlled state is associated with a rise in FeNO level [11]. Further, maintenance doses of inhaled corticosteroids can be reduced without loss of asthma control on the basis of FeNO level [12,13]. However, its value in eosinophilic parenchymal lung disease is unknown because inducible nitric oxide synthetase, the major source of FeNO, is usually found in airway epithelium [14].

Recently, our group reported that FeNO level is significantly higher in patients with acute eosinophilic pneumonia (AEP) than in those without AEP and decreases during corticosteroid treatment, strongly suggesting that FeNO level increases in eosinophilic parenchymal lung diseases [15]. Further, FeNO level is lower in patients with stable bronchiectasis than in those with asthma or chronic obstructive lung disease, implying that FeNO has no role in neutrophilic airway inflammation [16]. In this study, we explored the significance of FeNO in the diagnosis and management of CEP, an eosinophilic lung parenchymal disease, by investigating its relationships with peripheral eosinophilia and the clinical state of CEP and its validity for predicting exacerbation of CEP.

## Methods

### Study design and definitions

This retrospective cohort study was conducted at Seoul National University Bundang Hospital between November 2011 and October 2012. The Institutional Review Board approved the study protocol and waived the need for informed consent from patients (B-1210-174-105).

Diagnosis of CEP was based on the following criteria: (i) pulmonary opacities with peripheral predominance on chest radiography; (ii) peripheral eosinophilia ≥ 1000 cells/μL and/or alveolar eosinophilia ≥ 40% of the eosinophil count in BAL fluid; (iii) respiratory symptoms for over 2 weeks; and (iv) exclusion of known causes of eosinophilic pneumonia (parasitic infection, drugs, or allergic bronchopulmonary aspergillosis), eosinophilic granulomatosis with polyangiitis (Churg–Strauss syndrome), and hypereosinophilic syndrome [1].

Exacerbation was defined as reappearance of characteristic infiltrates on chest radiography, recurrence of typical clinical features, and increasing peripheral eosinophilia. Uncontrolled CEP was defined as administration or increasing dosage of corticosteroids due to diagnosis or exacerbation of CEP. Controlled CEP was defined as absence of symptoms regardless of corticosteroid dose.

### Measurements

At each visit during the 1-year study period, we assessed symptoms, chest radiographic findings, peripheral eosinophil count and percentage, and FeNO level. The recall interval was individualized according to the clinical state: most patients were reexamined every 2–3 months, but some patients with uncontrolled CEP were recalled before the scheduled appointment. Change in FeNO levels between visits was calculated at every assessment point, as follows: ΔFeNO = FeNO_*n*_ − FeNO_*n*−1_, where *n* and *n* − 1 represent the *n*-th and preceding visits, respectively. Changes in peripheral eosinophil count (Δeosinophil count) and percentage (Δeosinophil percentage) were similarly calculated.

FeNO level was measured by using a NIOX MINO monitor (Aerocrine AB, Solna, Sweden), without the nose clip, at an exhalation flow rate of 50 mL/s, according to the American Thoracic Society (ATS)/European Respiratory Society (ERS) recommendation [17]. A FeNO level > 50 ppb was considered indicative of eosinophilic inflammation and responsiveness to corticosteroids in symptomatic patients and an increase over 10 ppb suggested a significant ΔFeNO value [13].

### Treatment

The parameters except FeNO level were used to tailor the corticosteroid treatment. The initial regimen for patients with newly diagnosed or uncontrolled CEP was 0.5 mg/kg/day of prednisolone. The dose was gradually tapered according to the clinical state. Patients with controlled CEP generally received a maintenance dose of 2.5- to 5-mg prednisolone daily. If no exacerbation event occurred during 3 months of maintenance treatment, the medication was discontinued. If symptom aggravation, reappearance of radiopacities, and peripheral eosinophilia were noted, suggestive of uncontrolled CEP, the dosage was increased up to 0.5 mg/kg/day.

### Statistical analysis

Data are presented as median (interquartile range [IQR]) values or number (%) of patients. FeNO levels and peripheral eosinophil counts or percentages were analyzed with Pearson correlation analysis. Continuous variables were analyzed by using the Mann–Whitney *U*-test. The Wilcoxon signed-rank test was used to evaluate parametric differences during an exacerbation event and after corticosteroid administration. Receiver operating characteristic (ROC) curve analysis was used to determine the parametric values that best predicted exacerbation of CEP. *P* < 0.05 was considered to be statistically significant. All analyses were performed by using SPSS for Windows (version 18.0, SPSS, Inc., Chicago, IL, USA).

## Results

### Baseline characteristics

Eighteen patients (10 men) were enrolled in the study; fifteen patients had been diagnosed before the study began. The median age was 56 (41–68) years (Table 1). One patient (5.6%) was a current smoker, and 17 patients (94.4%) had never smoked or had stopped smoking. The most common symptom was cough (*n* = 15), followed by sputum production (*n* = 13). One patient (5.6%) had a history of pulmonary tuberculosis. The median peripheral eosinophil percentage was 21.2% (11.6–42.3%) of the total leukocyte count and median peripheral eosinophil count was 1543 (771–4034) cells/μL. Seven patients underwent BAL for diagnosis of CEP. The median eosinophil percentage in BAL fluid was 47% (15–65%).

**Table 1:** Baseline characteristics of the 18 patients with CEP at diagnosis

**Variable**	Value*	**Number reported/tested**
Age (years)	56 (41–68)	18
Disease duration (months)	19.1 (9.9–28.7)	18
Gender		18
Female	8 (44.4)	
Male	10 (55.6)	
Smoking status		18
Current smoker	1 (5.6)	
Ex-smoker	4 (22.2)	
Pack-year history	25 (20–40)	
Symptoms or signs		18
Cough	15 (83.3)	
Sputum	13 (72.2)	
Dyspnea	5 (27.8)	
Fever	2 (11.1)	
Wheezing	4 (22.2)	
Crackle	3 (16.7)	
History of tuberculosis	1 (5.6)	18
Laboratory findings		
White blood count (cells/μL)	8060 (7470–9660)	18
Peripheral eosinophil percentage (%)	21.2 (11.6–42.3)	18
Peripheral eosinophil count (cells/μL)	1543 (771–4034)	18
Eosinophil percentage in BAL fluid (%)	47 (15–65)	7
C-reactive protein (mg/dL)	1.5 (0.30–3.27)	15
Aspartate aminotransferase (IU/L)	21 (17–24)	17
Alanine aminotransferase (IU/L)	17 (12–24)	17
Blood urea nitrogen (mg/dL)	10 (8–11)	17
Creatinine (mg/dL)	0.84 (0.66–0.98)	17
Spirometric results		
Forced expiratory volume in 1 s/forced vital capacity ratio	76.0 (62–82)	15
% predicted forced expiratory volume in 1 s	87.0 (67–100)	15
% predicted forced vital capacity	86.0 (72–97)	15

*Data represent median (IQR) or number of patients (%).

### Clinical course

**Figure 1:**
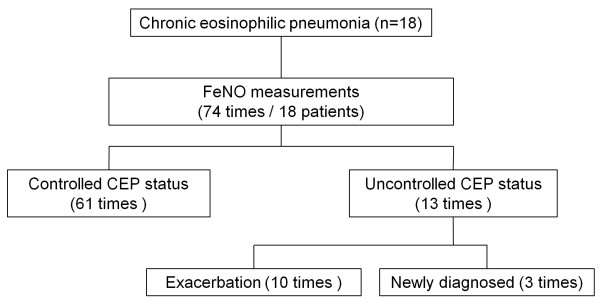
Schematic of the study.

Seven patients (38.9%) had controlled CEP throughout the study. Ten exacerbation events occurred in nine patients (50%), with one patient experiencing two episodes. In total, 74 FeNO measurements were obtained from the 18 patients, including 10 measurements during exacerbation events and three at diagnosis. Therefore, 13 FeNO measurements were obtained during uncontrolled CEP (Figure 1). Median time interval between patient visits was 56 days (IQR 28–77). Median number of visits that were attended by the patients was 4 (IQR 4–5).

### Relationship of FeNO and peripheral eosinophilia

**Figure 2:**
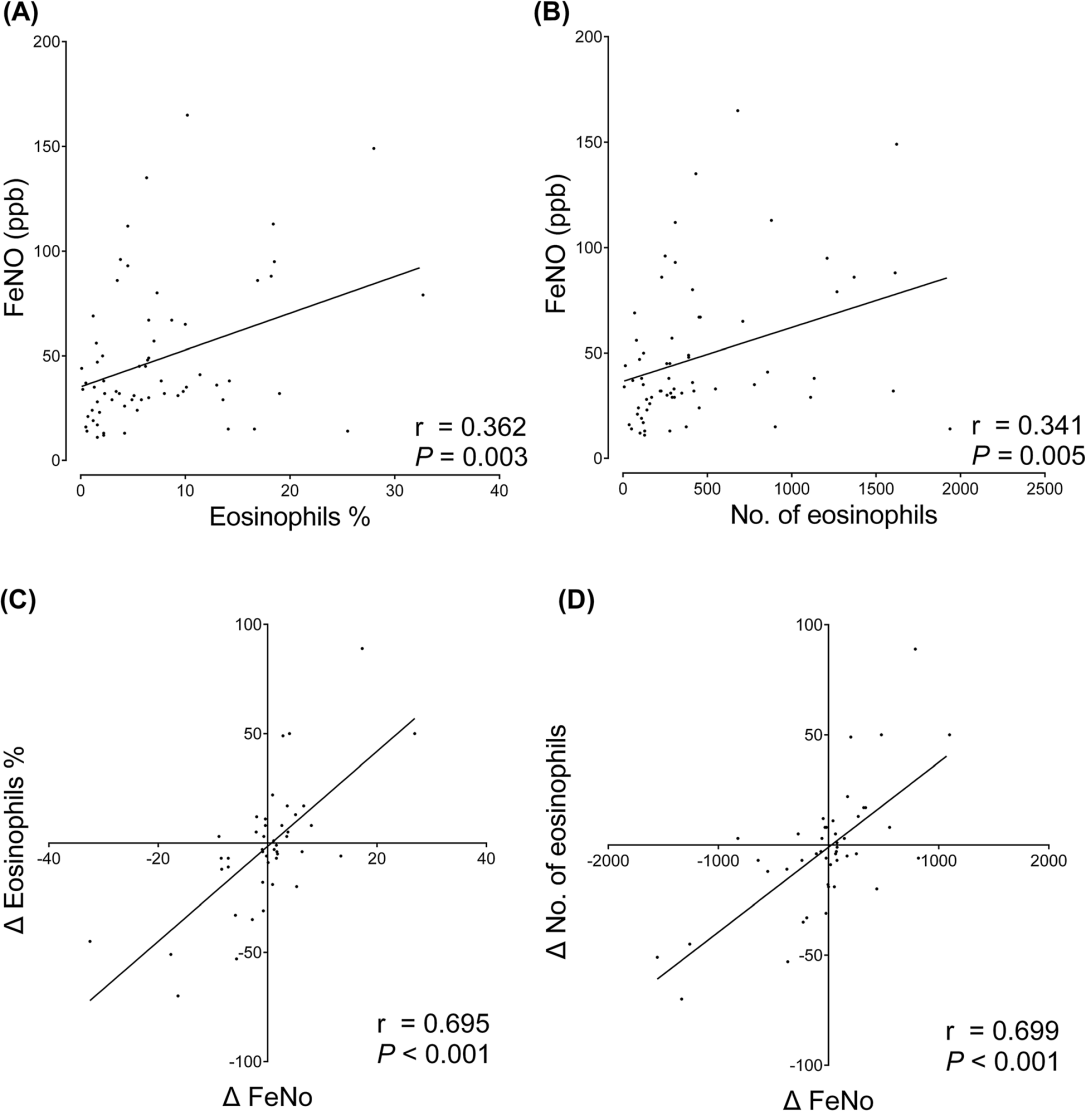
**Relationship of FeNO level and peripheral eosinophilia in CEP.** Scattergrams of FeNO level against peripheral eosinophil percentage **(A)** and count **(B)** as well as ΔFeNO against Δeosinophil percentage **(C)** and Δeosinophil count **(D)** at every assessment point are shown.

The median FeNO value, peripheral eosinophil percentage, and peripheral eosinophil count were 37 (11–165) ppb, 5.1% (0.0–32.7%), and 283 (0–1938) cell/μL, respectively. FeNO level was positively but weakly correlated with peripheral eosinophil percentage (*r* = 0.362, *P* = 0.003) and count (*r* = 0.341, *P* = 0.005). ΔFeNO was positively and moderately correlated with Δeosinophil percentage (*r* = 0.695, *P* < 0.001) and Δeosinophil count (*r* = 0.699, *P* < 0.001) (Figure 2).

### Relationship of FeNO and clinical state

**Figure 3:**
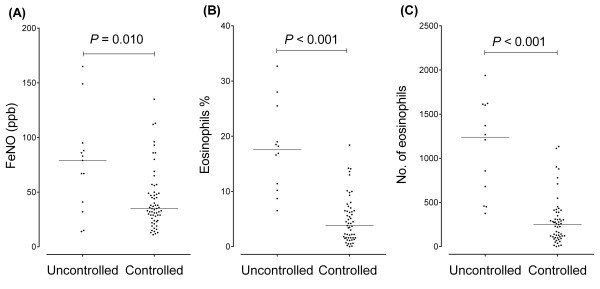
**Parametric changes according to the clinical state of CEP.** FeNO levels **(A)**, peripheral eosinophil percentages **(B)**, and peripheral eosinophil counts **(C)** in the controlled and uncontrolled states of CEP are shown.

The median FeNO levels were 79 (41–88) and 35 (26–49) ppb in uncontrolled (13/74 measurements) and controlled (61/74 measurements) CEP, respectively, showing a significant difference between the clinical states (*P* = 0.010). The median peripheral eosinophil percentage was also higher in the uncontrolled state (17.5% [10.8–22.3%] vs. 3.8% [1.6–6.5%]; *P* < 0.001). The peripheral eosinophil count showed a similar result (1239 [569–1608] cells/μL vs. 250 [110–389] cells/μL; *P* < 0.001) (Figure 3).

**Figure 4:**
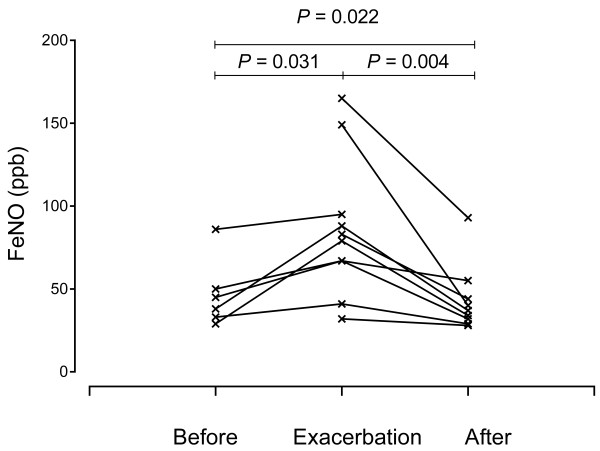
FeNO levels during exacerbation of CEP and after corticosteroid treatment.

In nine of the 10 exacerbation events, FeNO level was measured after corticosteroid administration. The median FeNO level significantly decreased after the treatment (81 [67–95] ppb vs. 37 [32–44] ppb; *P* = 0.004]. In five events, FeNO level was measured both before and after the episode. Significant changes in FeNO level was noted according to the clinical state of CEP (*P* = 0.022) (Figure 4). There is one current smoker. His FeNO was measured after one hour cessation of smoking according to previous recommendation because smoking may decrease FeNO level [18,19]. His FeNOs were measured two times in stable state (29 and 33 ppb) which were within controlled state IQR (26–49).

### Diagnostic accuracy of FeNO

**Figure 5:**
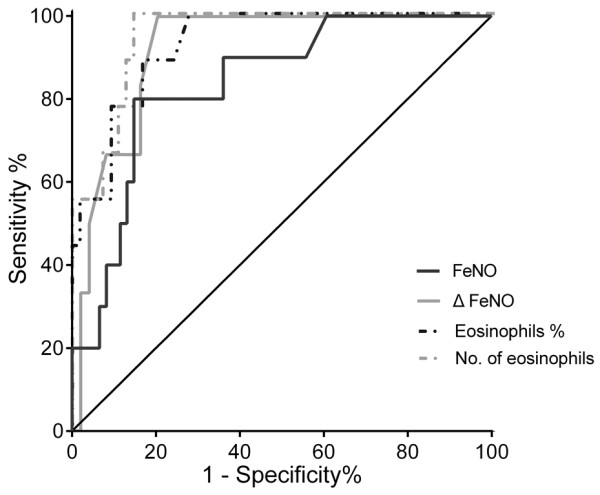
**ROC curves of the studied parameters for predicting exacerbation of CEP.** ROC curves of FeNO level (black solid line), ΔFeNO (gray solid line), peripheral eosinophil percentage (black dotted line), and peripheral eosinophil count (gray dashed-dotted line) are shown.

The area under the curve (AUC) of FeNO level was 0.835 (95% confidence interval = 0.716–0.954). At the cutoff level of 66 ppb, the sensitivity and specificity were 0.80 and 0.84, respectively. Further, the AUC of ΔFeNO was 0.918; at the cutoff value of 8.4 ppb, the sensitivity and specificity were 0.83 and 0.84, respectively (Figure 5 and Table 2). With regard to the ATS guidelines [20], the sensitivity and specificity at the 50-ppb cutoff level were 0.80 and 0.77, respectively, and those at the 10-ppb cutoff level of ΔFeNO were 0.67 and 0.86, respectively (Table 2).

**Table 2:** Diagnostic validity of the studied parameters for exacerbation of CEP

**Variable**	**AUC**	**Cutoff value**	**Sensitivity**	**Specificity**
FeNO level	0.835	66.0 ppb	0.80	0.84
		50.0 ppb*	0.80	0.77
ΔFeNO	0.918	8.4 ppb	0.83	0.84
		10.0 ppb**	0.67	0.86
Peripheral eosinophil percentage	0.906	8.4%	0.89	0.79
Peripheral eosinophil count	0.923	451 cells/μL	0.89	0.83

*FeNO level > 50 ppb is indicative of eosinophilic inflammation and corticosteroid responsiveness in symptomatic patients [20].

**ΔFeNO > 10 ppb indicates significant increase in FeNO level [20].

The peripheral eosinophil percentage of 8.4% showed the best sensitivity (0.89) and specificity (0.79) for predicting exacerbation of CEP (AUC = 0.906). Further, the peripheral eosinophil count of 451 cells/μL was the best cutoff value (AUC = 0.923; sensitivity = 0.89; specificity = 0.83) (Figure 5 and Table 2).

## Discussion

In this study, we evaluated FeNO as a potential marker of eosinophilic parenchymal inflammation and the clinical course of CEP. We found a moderate positive correlation between FeNO level and the degree of peripheral eosinophilia. Uncontrolled CEP was associated with a significantly higher FeNO level, and FeNO level increased during exacerbation events and decreased after corticosteroid treatment. To the best of our knowledge, this is the first study of FeNO in patients with CEP.

There are no clear diagnostic criteria for CEP. Most authors do not recommend histopathologic proof for establishing the diagnosis. Its diagnosis is based on suggestive clinical features, characteristic radiographic appearance, and peripheral eosinophilia [1,3,21]. We applied the Marchand et al. [1] criteria in this study. BAL fluid analysis may be helpful in diagnosis but is not a prerequisite [9,11]. The patients who did not undergo BAL not only met the diagnostic criteria but also demonstrated the clinical course of CEP.

CEP seems to show a pattern of waxing and waning frequently. Most patients experience exacerbation events when corticosteroid treatment is discontinued or tapered [1,2]. In previous long-term follow-up studies, 59–69% of the patients were still prescribed oral corticosteroid at the last follow-up visit [1,2]. In this study, 72.2% (13/18) used prednisolone at the last assessment point.

Numerous studies have demonstrated the relationships of FeNO with eosinophilic airway inflammation [20] and peripheral eosinophilia [22,23] in asthma. The positive correlation between FeNO level and peripheral eosinophilia in this study suggests that FeNO may reflect eosinophilic inflammation in CEP. Further, FeNO level increases during uncontrolled asthma and decreases during treatment with anti-inflammatory agents [6,24]; increase in FeNO level also predicts loss of asthma control [25]. In the present study, FeNO level showed a similar trend.

Peripheral eosinophil counts do not necessarily indicate the extent of eosinophilic involvement in affected tissue [26]. The present results show that FeNO measurement is not inferior to peripheral eosinophil percentage or count for monitoring eosinophilic inflammation in CEP. In some ways, it is more useful because the measurement method is completely noninvasive and easy to apply, and results are obtained immediately [20]. Moreover, the handheld FeNO monitor has the advantage of home-based use [27].

The FeNO level of 66.0 ppb showed the largest AUC with high sensitivity and specificity for predicting exacerbation of CEP. This value is near the ATS-recommended cutoff level (>50 ppb) [20], which also showed good sensitivity (80%) and specificity (77%). To account for each patient’s state of eosinophilic inflammation, we also evaluated ΔFeNO. The change in peripheral eosinophilia correlated well with ΔFeNO. Furthermore, the ΔFeNO value of 8.4 ppb showed good sensitivity and specificity for predicting exacerbation of CEP, similar to the ATS-recommended value of 10 ppb [20].

The present study has several limitations. First, all the FeNO measurements were combined because of the irregular assessment points in the small number of cases. However, the FeNO levels were simultaneously measured with the peripheral eosinophil and clinical parameters. Second, the 1-year follow-up duration is not enough to predict the long-term course of CEP. Third, the clinicians were aware of each patient’s FeNO levels, although they did not use them for tailoring the corticosteroid treatment. Fourth, FeNO levels of only three patients were measured at diagnosis of CEP. Additional FeNO data are needed to determine the cutoff value for diagnosis of CEP. Fifth, this retrospective study was conducted at a single center. Prospective multicenter clinical trials are required to analyze the association of symptoms, peripheral eosinophilia, and FeNO.

## Conclusions

FeNO may be a useful marker for monitoring eosinophilic parenchymal inflammation and determining the appropriate corticosteroid dose in CEP.

## Abbreviations

AEP: Acute eosinophilic pneumonia; AUC: Area under the curve; BAL: Bronchoalveolar lavage; CEP: Chronic eosinophilic pneumonia; FeNO: Fractional exhaled nitric oxide; IQR: Interquartile range; ppb: Particles per billion; ROC: Receiver operating characteristic.

## Competing interests

The authors declare that they have no competing interests.

## Authors’ contributions

JYP developed the study design, measured FeNO levels, analyzed the data, and drafted the manuscript. TL, HYL, YJL, JSP, YJC, HIY, and JHL selected and followed the patients and critically read the paper. CTL conceived the study, developed the study design, collected the data, and drafted and revised the manuscript. All the authors read and approved the final manuscript.

## Pre-publication history

The pre-publication history for this paper can be accessed here:

http://www.biomedcentral.com/1471-2466/14/81/prepub
